# The Pleasure of Being a Veterinarian1Paper read before the Pennsylvania State Veterinary Medical Association, March 4, 1896.

**Published:** 1897-06

**Authors:** J. C. Michener

**Affiliations:** Colmar, Pa.


### THE PLEASURE OF BEING A VETERINARIAN.[1]

By J. C. Michener, V.S.
COLMAR, PA.

Occupation helps to form character. Different callings bring into play and develop special faculties. As the business, so the man. A person may engage in a monotonous routine business, like a special part in a great factory, to which he becomes so accustomed as to act in an automatical manner, and become, as it were, a part of the machinery, exercising scarcely more thought than the mechanism that his hands direct. It is one of the charms and advantages of our profession that the practitioner must think and act for himself. It is preëminently an intellectual occupation, and one's success is measured by his powers of observation and ability to determine the requirements of the manifold conditions in which he finds his patients. The practitioner soon gains a keen realization of the necessity of being wideawake to take in the situation and anticipate the conditions that will arise, and be ready to meet them as the game-player. It is when he becomes equally fascinated, and when every failure makes him more determined, that he is on the road to success and honor, instead of the disaster and ruin which overtake the gambler. As practitioners, we know the difficulties encountered, the great care and close observation required to make correct diagnosis and prognosis, control our patients, and satisfy our patrons in a business point of view.

In real abilities required we lose nothing when put in comparison with the noblest of all professions. Our patients suffer from equally numerous and intricate diseases; require as skilful surgery; but, *unfortunately*, they are mute. We *must* learn the symptoms of diseases, the language of every attitude and movement. We do not *ask* to have a pain located and described, but make it out for ourselves. Put the human and veterinary practitioner in the field of the other, and we would be at least equally successful.

[1] Paper read before the Pennsylvania State Veterinary Medical Association, March 4, 1896.

Prognosis is one of our essential acquirements. We must tell at the onset the duration and result of the disease, the trouble and expense it will involve, the outcome of malformations, predispositions, freaks of nature, or odd behavior, in order to give our clients sound advice from a business point of view. This required knowledge is not of the horse alone, but of all living creatures over which man has control. Who can mention a business requiring a more diversified knowledge, more close observation and better judgment? Skill is always appreciated; we honor the person who can do something beyond the capabilities of ordinary mortals; hence our profession opens a field of rare usefulness, honor, and moderate profit, all of which lend rational abiding pleasure to our calling.

As you practitioners well know, it is not all fair sailing; the difficulties are great, and mistakes are censured unmercifully. Fortunate that it is so. It teaches us caution, develops our manhood, makes us self-reliant and strong. Our facilities for intercourse with our fellow men, the gratitude and friendship accorded us are among the substantial pleasures that fall to our lot. I imagine the country practitioner is in better touch and sympathy with his patron than his city brother. The farmer cares for his own stock; he receives the veterinarian as his guest; they become interested in each other, and a lasting friendship results. It is very pleasant and exhilarating to sit behind a good roadster and glide along amid the beauties of nature, and receive the friendly greeting of those one meets on the road. It is a great source of pride and pleasure that the veterinary art is rapidly gaining recognition and prominence among the professions, and that the time is growing short in which unworthy persons shall find employment because there are not enough qualified men to fill the public want.

We should all take pride in our business, and do our utmost to have a good class of men, thoroughly trained, in whose hands to place its keeping.

